# 

**Published:** 1857-09

**Authors:** 


No. IV.] SEPTEMBER. [Vol. XIII.

### BUFFALO

### MEDICAL JOURNAL

AND

### Monthly Review

OF

### MEDICAL AND SURGICAL SCIENCE.

SANFORD B. HUNT, M. D.,
EDITOR.

AUSTIN FLINT, Jr., M. D.,
ASSOCIATE EDITOR.

BUFFALO, N. Y.:
E. R. JEWETT & CO.'S STEAM PRESS.
Commercial Advertiser Buildings.

1857.

Price $2.00 in advance, or $2.50 at the end of three months.

